# Prognóstico da Doença Arterial Coronariana em Hospitais Públicos no Brasil: O Estudo ERICO e Uso do Conhecimento na Saúde Pública

**DOI:** 10.36660/abc.20210825

**Published:** 2021-11-01

**Affiliations:** 1 Universidade Federal de Minas Gerais Programa de Pós-Graduação em Saúde Pública Faculdade de Medicina Belo Horizonte MG Brasil Programa de Pós-Graduação em Saúde Pública, Faculdade de Medicina, Universidade Federal de Minas Gerais, Belo Horizonte, MG – Brasil; 2 Organização Internacional de Saúde Pública Vital Strategies Nova Iorque EUA Organização Internacional de Saúde Pública Vital Strategies, Nova Iorque – EUA

**Keywords:** Doença da Artéria Coronariana, Prognóstico, Hospitais Públicos, Epidemiologia, Saúde Pública, Fatores de Risco, COVID-19, Mortalidade

O grupo de doenças cardiovasculares (DCV) agrega as principais causas de morte no Brasil e no conjunto dos países em desenvolvimento.^1,2^ A doença isquêmica do coração (DIC) ou doença arterial coronariana (DAC) tem sido por muitos anos a principal causa de morte na população brasileira,^1^ com exceção do ano de 2020,^3^ quando a doença causada pelo novo coronavírus (COVID-19) foi a primeira causa de morte, seguida pela DIC. A DIC foi a principal causa de anos de vida perdidos na população brasileira em 2016.^4^

Em 2017, foi estimada uma prevalência de DAC de 1,75% (2.500.000 de indivíduos), na população brasileira maior que 20 anos.^1^ As maiores prevalências estavam nas regiões Sul e Sudeste, com taxa de mortalidade padronizada decrescente, mas com aumento da prevalência desde 1990.^1^ Com estimativa de incidência de cerca de 121 mil casos por ano^1^ em 2017, a DAC tem sido um importante problema de saúde pública no país.

O estudo ERICO,^5^ coorte de pacientes com episódio de síndrome coronária aguda (SCA) atendidos em um hospital secundário, entre outros estudos, é peça importante na produção do conhecimento sobre prognóstico de curto e longo prazos de pacientes em cuidado secundário e DCA.

Uma pergunta que está presente diante dos pacientes com SCA é qual a melhor intervenção, quais as evidências, qual o prognóstico. Como informar aos pacientes e as famílias sobre as chances da sobrevida de longo prazo se ainda não há um conhecimento consolidado, muitas questões ainda não respondidas para a realidade brasileira, como, por exemplo, impacto dos determinantes sociais^6^ no prognóstico. Quais as evidências sobre o melhor tratamento?

A estatística cardiovascular^1^ publicada em 2020 revelou que “*foram realizadas 78.575 angioplastias coronárias pelo SUS em 2018, com mortalidade hospitalar de 2,96% e média de permanência hospitalar de 4,5 dias*”. Com esse número de angioplastias, a possibilidade de uso das melhores evidências para informar sobre o melhor cuidado e procedimentos, aumentam as chances de beneficiar não somente o paciente indivíduo, mas principalmente, os milhares de doentes com SCA incidente, reduzindo a mortalidade populacional e melhorando a qualidade de vida. 10% das hospitalizações^7^ no SUS foram por DCV em 2019.

O uso da tecnologia para o diagnóstico e tratamento durante uma manifestação aguda da doença (particularmente acidente vascular cerebral ou infarto agudo do miocárdio [IAM]) foi fundamental em muitos países para redução de mortes e prolongamento da vida quando a DCV se manifesta.^8^

Cristina-Bruno et al.,^9^ revelam no artigo que “*não só os pacientes com doença de múltiplos vasos como também os com doença de um vaso tiveram alto risco de mortalidade no longo prazo pós-SCA. Esses achados destacam a importância de se ter uma abordagem melhor no tratamento e no controle de FRC, mesmo em indivíduos com risco aparentemente baixo, atendidos em cuidado secundário*”.

O surgimento e o rápido crescimento de fatores de risco cardiovascular (FRC) em países em desenvolvimento são responsáveis pelo forte aumento na morbidade/mortalidade relacionada a DIC nas últimas décadas, trazendo a necessidade de um plano de controle epidemiológico destinado a prevenir DCV em países em desenvolvimento.^4,5,7,10^

A maior mortalidade por DAC está relacionada com um menor nível socioeconômico,^5^ países de maior renda têm taxa de mortalidade menor que países de média renda.^1,4^ Os novos tratamentos da DAC com uso de novas tecnologias têm reduzido a mortalidade, mas não podem reduzir a carga da doença e a perda da saúde^1,4^ associada com a DAC. Os fatores de risco como obesidade, dieta, tabagismo e sedentarismo têm aumentado o risco para o desenvolvimento da doença.^1,2,4–6,9^ A associação crescente DAC e diabetes, vem contribuindo para aumento do risco de óbito.^11–13^

A linha de base do estudo ERICO^6^ mostrou “uma *média de idade foi de 62,7 anos, 58,5% homens e 77,4% tinham 8 anos ou menos de estudo. Os fatores de risco cardiovascular mais comuns foram hipertensão (76%) e sedentarismo (73,4%). Apenas 29,2% tinham história prévia de doença coronariana*”.^6^

No período 1990-2017, a prevalência de DAC aumentou nos dois sexos (de 1,08% para 1,75%), de maneira mais proeminente em homens que em mulheres, aumentando com o envelhecimento da população.^1,8^

Considerando a importância do tratamento da morbidade cardiovascular e seus eventos agudos, a tendência de redução da mortalidade por DAC e, o consequente, aumento da sobrevida dos pacientes com SCA e obstrução coronariana, trouxe a necessidade de elevar o conhecimento sobre o tratamento,^9^ o melhor uso das informações clínicas para o prognóstico^8,13^ e prevenção dos FRC.^4^ Para tanto seria fundamental conhecer as práticas dos profissionais de saúde e seu nível de adesão às recomendações de boas práticas.^14^

Assim, conhecer com mais profundidade, produzir evidências, buscar impacto a nível populacional^8,13,14^ e, ao mesmo tempo, colocar no centro do debate sobre redução da prevalência e incidência de SCA e DAC as políticas de saúde pública^8^ para enfrentamento do crescente aumento dos FRC, como a forma mais efetiva para reduzir as perdas de saúde e os anos de vida perdidos devido a DAC.^4^

## References

[1] Oliveira GMM, Brant LCC, Polanczyk CA, Biolo A, Nascimento BR, Malta DC, et al. Estatística Cardiovascular – Brasil 2020. Arq Bras Cardiol. 2020; 115(3):308-439. 10.36660/abc.20200812 PMC9363085 33027364

[2] Ribeiro ALP, Duncan BB, Brant LCC, Lotufo PA, Mill JG, Barreto SM. Cardiovascular Health in Brazil Trends and Perspectives. Circulation. 2016; 133(4):422–33. 10.1161/CIRCULATIONAHA.114.008727 26811272

[3] COVID-19 Results briefing Brazil, [acessado em setembro de 2021] Disponível: http://www.healthdata.org/sites/default/files/covid_briefs/135_briefing_Brazil.pdf

[4] Marinho F, Passos VMA, Malta DC, França EB, Abreu DMX, Araújo V, et al. Burden of disease in Brazil, 1990-2016: a systematic subnational analysis for the Global Burden of Disease Study 2016. Lancet.2018;392(10149):760-75. 10.1016/S0140-6736(18)31221-2 PMC6123514 30037735

[5] Abreu SLL, França de Abreu JDM, Branco MRFC, Santos AM. Óbitos Intra e Extra-Hospitalares por Infarto Agudo do Miocárdio nas Capitais Brasileiras. Arq Bras Cardiol. 2021; 117(2):319-26. 10.36660/abc.20200043 PMC8395787 34495227

[6] Goulart AC, Santos IS, Sitnik D, Staniak KL, Fedeli LM, Pastore CA, et al. Design and baseline characteristics of a coronary heart disease prospective cohort: two-year experience from the strategy of registry of acute coronary syndrome study (ERICO study). CLINICS 2013; 68(3):431-4 10.6061/clinics/2013(03)RC02 PMC3611751 23644870

[7] Marmot M, Bell R. Fair society, healthy lives. Public Health, 2012; 26(1): S4-S10. 10.1016/j.puhe.2012.05.014 22784581

[8] Ramires JA. Implementação de Programas de Melhoria de Qualidade Assistencial. Arq Bras Cardiol. 2020; 115(1):100-1. 10.36660/abc.20200679 PMC8384331 32813827

[9] Bruno CT, Bittencourt MS, Quidim AVL, Santos I, Lotufo P, Bensenor I, Goulart A. O Prognóstico da Doença Arterial Coronariana em um Hospital Público no Brasil: Achado do Estudo ERICO. Arq Bras Cardiol. 2021; 117(5):978-985. 10.36660/abc.20200399 PMC8682093 34644783

[10] Verdier F, Fourcade L. Changes in cardiovascular risk factors in developing countries. Medecine Tropicale: Revue du Corps de Santé Colonial. 2007; 67(6):552-8. PMID: 18300515. 18300515

[11] Siqueira AFA, Almeida-Pititto B, Ferreira SRG. Doença cardiovascular no diabetes mellitus: análise dos fatores de risco clássicos e não-clássicos. Arq Bras Endocrinol Metab. 2007;51(2) 10.1590/s0004-27302007000200014 17505632

[12] Santos IS, Goulart AC, Brandão RM, Santos RCO, Bittencourt MS, Sitnik D, et al. Mortalidade em um Ano após Evento Coronário Agudo e seus Preditores Clínicos: O estudo ERICO. Arq Bras Cardiol. 2015; 105(1):53-64. 10.5935/abc.20150044 PMC4523288 25993485

[13] Viana MS, Lopes F, Cerqueira-Junior AMS, Suerdieck JG, Barcelos da Silva A, Souza TM, et al. Valor Prognóstico Incremental da Incorporação de Dados Clínicos à Anatomia Coronária em Síndromes Coronarianas Agudas: Escore SYNTAX-GRACE. Arq Bras Cardiol. 2017; 109(6):527-32. 10.5935/abc.20170160 PMC5783433 29160388

[15] Taniguchi FP, Bernardez-Pereira S, Silva SA, Ribeiro ALP, Morgan L, Curtis AB, et al. Implementação do Programa Boas Práticas em Cardiologia adaptado do Get With The Guidelines® em Hospitais Brasileiros: Desenho do Estudo e Fundamento. Arq Bras Cardiol. 2020; 115(1):92-9. 10.36660/abc.20190393 PMC8384317 32187286

