# Supplementary material for: Prognóstico da Doença Arterial Coronariana em Hospitais Públicos no Brasil: O Estudo ERICO e Uso do Conhecimento na Saúde Pública
Source: Arq Bras Cardiol. 2021 Nov 1;117(5):986–7. [Article in Portuguese] doi: 10.36660/abc.20210825 (PMC8682102; doi:10.36660/abc.20210825)
Supplement: Translation

# Translation: Prognosis of Coronary Artery Disease in Public Hospitals in Brazil: The ERICO Study and the Application of Knowledge in Public Health

**Affiliations:** 1 Belo Horizonte MG Brazil Programa de Pós-Graduação em Saúde Pública, Faculdade de Medicina, Universidade Federal de Minas Gerais, Belo Horizonte, MG – Brazil; 2 New York USA Organização Internacional de Saúde Pública Vital Strategies, New York - USA

**Keywords:** Coronary Artery Disease, Prognosis, Hospitals, Public, Epidemiology, Public Health, Risk Factors, COVID-19, Mortality

The group of cardiovascular diseases (CVD) includes the leading causes of death in Brazil and in all developing countries.^1,2^ Ischemic heart disease (IHD) or coronary artery disease (CAD) has been the leading cause of death for many years in the Brazilian population,^1^ with the exception of the year 2020,^3^ when the disease caused by the novel coronavirus (COVID-19) was the leading cause of death, followed by IHD. IHD was the main cause of years of life lost in the Brazilian population in 2016.^4^

In 2017, the prevalence of CAD was estimated to be of 1.75% (2,500,000 individuals) in the Brazilian population over 20 years of age.^1^ The highest prevalence was found in the South and Southeast Regions, with a decreasing standardized mortality rate, but an increased prevalence, since 1990.^1^ With an estimated incidence of about 121,000 cases per year in 2017,^1^ CAD has been an important public health problem in Brazil.

The ERICO study,^5^ a cohort of patients with episodes of acute coronary syndrome (ACS) who were treated at a secondary hospital, among other studies, is an important element in the production of knowledge about the short- and long-term prognosis of patients receiving secondary care and CAD.

Questions that need to be asked for patients with ACS include the following: What is the best intervention, what is the evidence, and what is the prognosis? How are we to inform patients and families about the chances of long-term survival if there is still no consolidated knowledge? Many questions have yet to be answered in the Brazilian context, for example, the impact of social determinants^6^ on prognosis. What is the evidence regarding the best treatment?

Cardiovascular statistics^1^ published in 2020 revealed that “78,575 coronary angioplasties were performed by the SUS in 2018, with hospital mortality of 2.96% and average hospital stay of 4.5 days.” With this number of angioplasties, the possibility of applying the best evidence to provide information about the best care and procedures increases the chances of benefiting not only individual patients, but the thousands of patients with ACS, thus reducing population mortality and improving quality of life. In 2019, 10% of hospitalizations in the SUS were due to CVD.^7^

The use of technology for diagnosis and treatment during an acute manifestation of the disease (particularly stroke or acute myocardial infarction) has been instrumental in many countries for reducing deaths and prolonging life when CVD manifests.^8^

In their article, Bruno et al.,^9^ reveal that, “Not only patients with multiple vessel disease, but also those with single vessel disease had a high risk of long-term post-ACS mortality. These findings highlight the importance of having a better approach to treatment and control of cardiovascular risk factors, even in individuals with apparently low risks, who are treated in secondary care.”

The emergence and rapid growth of cardiovascular risk factors in developing countries are responsible for the prominent increase in morbidity and mortality related to IHD in recent decades, bringing about the need for an epidemiological control plan, with the aim of preventing CVD in developing countries.^4,5,7,10^

Greater mortality due to CAD is related to lower socioeconomic level,^5^ and higher income countries have lower mortality rates than middle-income countries.^1,4^ New treatments for CAD with the use of new technologies have reduced mortality, but they cannot reduce the disease burden and the loss of health^1,4^ associated with CAD. Risk factors, such as obesity, diet, tobacco use, and sedentary lifestyle, have increased the risk of developing the disease.^1,2,4–6,9^ The growing association of CAD and diabetes has contributed to an increased risk of death.^11–13^

The baseline of the ERICO study^6^ showed that, “Average age was 62.7 years; 58.5% were men, and 77.4% had 8 years of schooling or less. The most common cardiovascular risk factors were hypertension (76%) and sedentary lifestyle (73.4%). Only 29.2% had prior history of coronary disease.”^6^

During the period from 1990 to 2017, the prevalence of CAD increased in both sexes (from 1.08% to 1.75%), more prominently in men than in women, increasing with the aging of the population.^1,8^

Considering the importance of treating cardiovascular morbidity and its acute events, the trend of reduced mortality due to CAD and, consequently, the increased survival of patients with ACS and coronary obstruction have made it necessary to enhance knowledge about treatment,^9^ better use of clinical information for prognosis,^8,13^ and prevention of cardiovascular risk factors.^4^ It is, therefore, fundamental to understand health professionals’ practice and their level of adherence to good practice recommendations.^14^

Thus, understanding more in depth, producing evidence, seeking impact on the population level,^8,13,14^ and, at the same time, placing public health policies at the center of the debate on reducing the prevalence and incidence of ACS and CAD^8^ in order to face the growing increase in cardiovascular risk factors are the most effective way to reduce health losses and lost years of life due to CAD.^4^
